# The JNK Signaling Pathway in Inflammatory Skin Disorders and Cancer †

**DOI:** 10.3390/cells9040857

**Published:** 2020-04-02

**Authors:** Manel B. Hammouda, Amy E. Ford, Yuan Liu, Jennifer Y. Zhang

**Affiliations:** 1 Department of Dermatology, Duke University Medical Center, Durham, NC 27710, USA; manel.ben.hammouda@duke.edu (M.B.H.); amy.e.ford@duke.edu (A.E.F.); yuan.liu578@duke.edu (Y.L.); 2 Department of Pathology, Duke University Medical Center, Durham, NC 27710, USA

**Keywords:** JNK, skin inflammation, keratinocytes, BCC, SCC, melanoma, psoriasis, fibrosis, scleroderma

## Abstract

The c-Jun N-terminal kinases (JNKs), with its members JNK1, JNK2, and JNK3, is a subfamily of (MAPK) mitogen-activated protein kinases. JNK signaling regulates a wide range of cellular processes, including cell proliferation, differentiation, survival, apoptosis, and inflammation. Dysregulation of JNK pathway is associated with a wide range of immune disorders and cancer. Our objective is to provide a review of JNK proteins and their upstream regulators and downstream effector molecules in common skin disorders, including psoriasis, dermal fibrosis, scleroderma, basal cell carcinoma (BCC), squamous cell carcinoma (SCC), and melanoma.

## 1. The c-Jun N-Terminal Kinase (JNK) Signaling Pathway

### 1.1. JNK Pathway Components

JNK, also known as stress-activated protein kinases (SAPK), represents a subfamily of the canonical MAPK signal transduction pathway [1], which along with cyclin-dependent kinases (CDKs), glycogen synthase kinase 3 (GSK3), and CDK-like kinases (CLKs), constitutes a larger family referred to as the CMGC Ser/Thr group kinases [1,2,3]. JNK proteins, JNK1, JNK2, and JNK3, are encoded by three separate genes Mapk8, Mapk9, and Mapk10, respectively [4]. Each is alternatively spliced to create at least ten variants that were detected by Western blotting at approximately 46 kDa (e.g., JNK1α1 and JNK1β1) and 55 kDa (e.g., JNK1α2 and JNK1β2) molecular weights [5].

**Figure 1:**
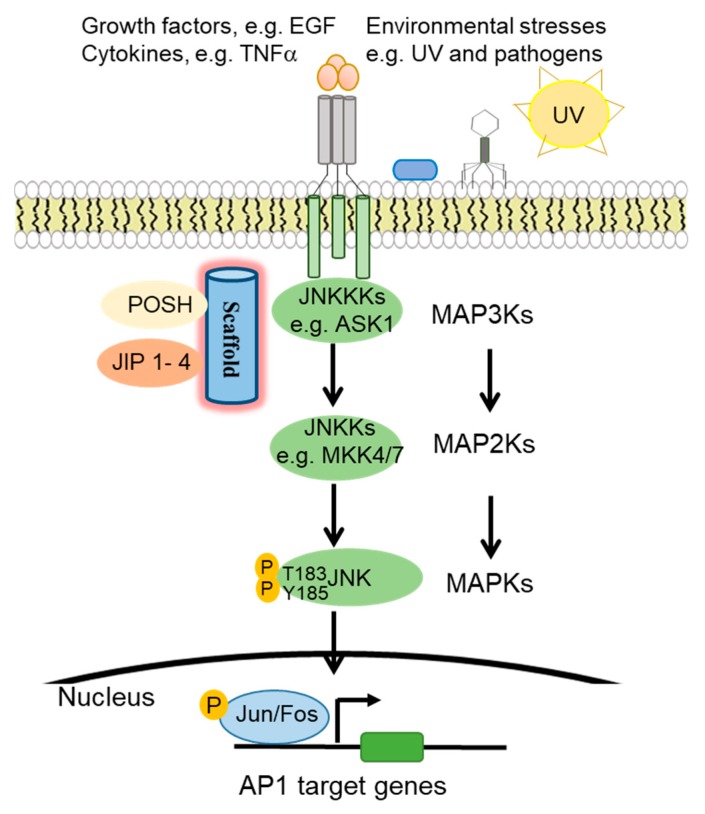
c-Jun N-terminal kinase (JNK) signaling pathway activation. In response to environmental stresses, growth factor, and cytokines, JNKKKs such as ASK1 phosphorylates JNKKs, specifically MKK4 and MKK7, which then activate JNK and finally the transcription factor activator protein-1 (AP1) family proteins.

JNK proteins are highly responsive to a diverse array of cellular stimuli, including inflammatory cytokines, growth factors, UV radiation, bacterial, and viral infections, heat shock, and osmotic and genotoxic stresses [6,7,8,9] (Figure 1). JNK is activated by JNKKs (JNK kinases), which in turn is regulated by JNKKKs (JNK kinase kinases) [10]. Specifically, JNK is activated by upstream MAPK2K (MKK4 and MKK7) via phosphorylation of the threonine and tyrosine residues of the conserved ThrProTyr (TPY) motif [11,12,13]. MAPK2Ks are subject to regulation by further upstream MAP3K and MAP4K proteins, as well as scaffold proteins such as the JNK interacting proteins (JIP1, JIP2, and JIP3) [14], SH3 proteins (e.g., POSH) [15], and the IκB kinase complex-associated protein (IKAP) [11,16,17]. Upon activation, JNK phosphorylates downstream target proteins such as the transcription factor activator protein-1 (AP1) family proteins, activating *transcription factors* (ATF), and (ETS Like-1 protein) Elk1 [13].

### 1.2. JNK Regulation of Cell Cycle Progression

**Figure 2:**
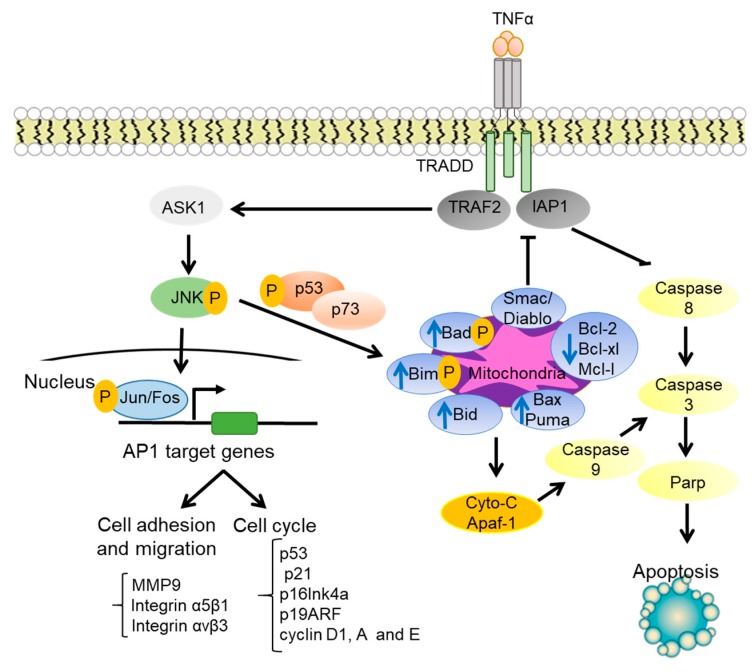
JNK regulation of cell cycle progression, cell adhesion, and cell apoptosis. In response to extracellular cytokines such as TNFα, JNK induces the phosphorylation of p53 and activation of p73. This process leads to the upregulation of the proapoptotic proteins (e.g., Bad, Bim, Bid, Bax, Puma) and Smac/Diablo and the downregulation of the antiapoptotic proteins (e.g., Bcl-2, Bcl-x, and Mcl-1). In parallel, JNK regulates cell cycle, cell adhesion, and cell migration through the activation of AP1 target genes.

The JNK signaling pathway mediates a wide range of cellular processes, including cell proliferation, survival, and migration [4,18,19], as well as cell apoptosis, senescence, and stress responses [20,21] (Figure 2). Genetic inactivation of MKK7 causes premature senescence and mouse embryonic fibroblast cell growth arrest in G2/M [21]. JNK regulates G1 cell cycle progression and G2/M transition. Activation of JNK occurs at the G2/M transition in Jurkat cells [22]. Activated JNK was also found localized in the centromeres during early S-phase to late anaphase with peak activity at metaphase in human HeLa cervical carcinoma cells [23]. Consistently, c-Jun is phosphorylated during mitosis and the early portion of the G1 phase [24]. JNK is also found to promote mitosis through Aurora B Kinase [25]. JNK inhibition by pharmacological or small interfering (siRNA)-mediated genetic approaches inhibits G2/M transition of NIH-3T3 fibroblasts and CIGC ovarian granulosa cells, which is attributed to the inhibition of Aurora B kinase and the subsequent loss of Histone-H3 (Serine 10) phosphorylation [18].

As an important JNK effector, AP1 functions as either homodimers containing two Jun proteins (c-Jun, JunB, and JunD) or heterodimers containing a Jun protein and a Fos protein (e.g., c-Fos, FosB, Fra1, and Fra1) [25]. AP1 regulates expression of many cell cycle regulators such as p53, p21(cip1/waf1), p16Ink4a, p19ARF, cyclin D1, cyclin A, and cyclin E [26,27]. Interestingly, AP1 proteins display differential roles in cell cycle regulation. For example, c-Jun activates Cyclin D1 promoter, whereas JunB suppresses it [24]. AP1 also plays a crucial role in the extracellular matrix (ECM) remodeling and promotes angiogenesis [28]. AP1 is induced by fibronectin and vitronectin via integrin α5β1/αvβ3-dependent JNK, Akt, and ERK signaling pathways, and consequently increases the expression of matrix metalloproteinase 9 (MMP9) in human umbilical vein endothelial cells, leading to enhanced angiogenesis [28].

### 1.3. JNK Regulation of Cell Survival and Apoptosis

JNK plays paradoxical roles in cell survival and apoptosis [29,30]. Jnk1−/−Jnk2−/− fibroblasts are sensitive to tumor necrosis factor (TNFα)-induced cell death, indicating that JNK promotes cell survival [30]. This study showed that JNK is required for expression of JunD transcription factor, which collaborates with NF-κB to promote the expression of pro-survival genes such as cIAP-2 in fibroblast [30]. JNK is also found to promote the survival of fibroblast-like synoviocytes in rheumatoid arthritis through downregulation of FoxO1 [31]. Other studies show that JNK acts synergistically with NF-κB and JAK/STAT to promote cell survival [32]. On the other hand, JNK is well-known to play an essential role in apoptosis [10,33,34]. JNK directly targets mitochondria through the phosphorylation of Bad and Bim, and these pro-apoptotic proteins antagonize the activity of anti-apoptotic proteins such as Bcl-2 and Bcl-xL [10]. In addition, JNK stimulates the release of cytochrome c (Cyt C) through a Bid-Bax-dependent mechanism, leading to the formation of apoptosomes consisting of Cyt C, caspase-9, and Apaf-1 and consequently the activation of Caspase 9-dependent apoptosis [10]. Moreover, JNK inhibits TRAF2/IAP1 signaling by induction of the release of Smac/Diablo from mitochondria and consequent activation of caspase 8 [10]. Inositol-requiring transmembrane kinase/endoribonuclease 1α (IRE1α) recruits TRAF2, which then activates ASK1 and JNK, leading to subsequent inhibition of anti-apoptotic proteins such as Bcl-2, Bcl-xl, and Mcl-1 [35]. In response to DNA damage, JNK mediates apoptosis by phosphorylation of p53, stabilization of p73, and p53/p73-dependent expression of Bax and Puma pro-apoptotic molecules [10,33,34,36].

## 2. JNK Signaling in Immunological Skin Disorders

### 2.1. JNK Regulation of Immune Responses

Immune responses are often divided into Th1, Th2, and Th17 T helper lymphocyte immunity [37]. Th1 cells release IL-2, interferon (IFN)-γ [38], and tumor necrosis factor (TNF)-β cytokines [39] and mediates responses against viral and bacterial infections and elimination of cancer cells [40]. Th2 cells produce interleukin IL-4, IL-5, IL-10, IL-13, and IL-33, and mediate humoral responses, B cell activation, and antibody production. Th17 cells secrete IL-17, IL-6, IL-22, and TNF-α. Th2 and Th17 play crucial roles in tissue repair and regeneration [41]. The balance between Th1 and Th2 immune responses is crucial for normal tissue homeostasis [41]; this is known as the Th1 and Th2 paradigm.

JNK plays a significant role in the innate and adaptive immune responses [42,43,44,45,46,47]. JNK activation promotes apoptosis of developing thymocytes and regulates T-cell differentiation and survival [45,48]. The Src-family tyrosine kinase Lck is associated with the CD4 and CD8 co-receptors. Lck expression is reduced in Th2 cells compared Th1 cells; Ectopic expression of Lck in Th2 cells led to increased expression of CD4 co-receptor and c-Jun phosphorylation at Serine 73 via concerted actions of JNK and ERK signaling [49], suggesting that Lck promotes Th1 polarization in part through a JNK-dependent process. JNK/c-Jun signaling is at least partly responsible for CD27-induced suppression of IL-17 and CCR6 expression and consequently reduced Th17 cell development and differentiation [50]. In CD8+ T cells, JNK1 is activated by POSH-mediated complex formation with MLK3, MKK7, and JIP-1, and thereby regulates cell proliferation and function [48]. In B-cells, JNK/c-Jun is activated by CD154-induced CD40 internalization in a JIP-dependent manner and regulates memory B-cell development [14].

As a critical regulator of immune cell differentiation and activation, JNK is implicated in many immune-related skin disorders. In this section, we will review the role of JNK in the development of psoriasis and dermal fibrosis.

### 2.2. JNK Contribution to Psoriasis

#### 2.2.1. Pathogenesis of Psoriasis

Psoriasis is one of the most common skin diseases affecting adults at approximately 2–3% of the world population [51]. Psoriasis is a chronic and dynamic disease where skin lesion morphology changes and advances over time, leading to a systemic disorder within the blood mirroring the high levels of cytokines and immune cells found in the skin lesions. Such severe systemic forms can extend to other organs, such as the musculoskeletal system in psoriatic arthritis [52]. Psoriasis involves dysregulation of epidermal cell proliferation and differentiation, blood vessel dilation, infiltration of T-cells and neutrophils, and an imbalance between CD4+ T effector cells, specifically the T helper (Th17) subset and regulatory T cells (Tregs) [52,53,54,55,56].

The pathogenesis of psoriasis is complex involving environmental triggers and genetic contributions [51]. Recent genome-wide association analyses have identified multiple psoriasis susceptibility loci (PSORS). Among these are PSORS1 which maps to HLA-Cw6 on the major histocompatibility chromosomal region 6p21.3, PSORS2 which maps to CARD14 gene in the chromosomal region 17q25-qter, and other genes involved in the regulation of interferon (IFN), NF-κβ and JNK signaling pathways [55,57,58,59,60]. Environmental triggers of psoriasis are less defined, but there is substantial evidence linking psoriasis to drug treatments and the microbiome [51,61,62].

#### 2.2.2. JNK and NF-κB Pathway Regulators in Psoriasis

CARD14 is highly expressed in epidermal keratinocytes, and its mutation is detected in both familial and non-familial psoriasis [63]. Overexpression of psoriasis-associated mutants of CARD14 in keratinocytes results in enhanced NF-κβ activation and upregulation of psoriasis-associated chemokines (e.g., CCL20 and IL-8) [63]. CARD14 shares structural similarity with CARD10 and CARD11, both of which act as scaffolds for signaling molecules such as the Mucosa-associated lymphoid tissue lymphoma translocation protein 1, MALT1, to mediate downstream signaling pathways, including JNK and NF-κβ [64,65]. Like CARD10 and CARD11, overexpression of the wild type or a shortened splice variant of CARD14 induces JNK/c-Jun phosphorylation, and c-Jun accumulation and CARD14 co-expression with MALT1 further enhances JNK activation [63]. These results indicate that psoriasis-associated CARD14 mutations induce inflammatory cytokines via MALT1-mediated aberrant activation of NF-κβ and JNK signaling pathways. In immune cells, JNK is shown to regulate FOXP3, an important transcription factor and, a master regulator of Treg development and function [66,67]. Mutations in FOXP3 impair nuclear localization and consequently loss-of-function of FOXP3 transcriptional activity [68]. High-level cytoplasmic retention of FOXP3 is associated with high IL-17 levels and disease severity [69]. Inhibition of JNK with SP600125 or siRNA knockdown in CD4+CD25+ T-cells resulted in increased cytoplasmic levels of FOXP3. FOXP3 nuclear translocation was mediated by an interaction with pc-Jun induced by JNK, and it is speculated that mutations in FOXP3 prevent its interaction with c-Jun and nuclear translocation, leading to Treg dysfunction and promotion of psoriasis [70].

#### 2.2.3. JNK Regulation of Dermal and Epidermal Interactions

Cysteine-rich angiogenic inducer 61 (Cyr61/CCN1) is a cell matrix chemokine found greatly enhanced in lesional skin of psoriatic patients [71,72]. CCN1 produced by fibroblasts of the imiquimod/IL-23-induced psoriasis mice aggravates epidermal hyperplasia and inflammation through JNK-mediated upregulation of CCL20 and IL-8 and subsequent recruitment of CCR6+ dendritic cells and T-cells into inflamed skin tissue [72]. The human β-defensin 2 (hβD-2) is an antimicrobial peptide produced by both keratinocyte and immune cells, and promotes keratinocyte proliferation and recruitment of Th1 and Th17 CCR6+ immune cells [73,74]. hβD-2 was found to act through JNK, MEK/ERK, and PI3K/Akt signaling pathways to increase T-cell production of Th1-associated cytokines, including IFNγ, TNFα, IL-1β, IL-6, and IL-22, and decrease expression of IL-17 [75]. In return, these cytokines modulate the expression of hβD-2, forming a positive feedback signaling loop. Serum hβD-2 levels were significantly increased in patients with psoriasis compared to healthy individuals, supporting a driver role of hβD-2 in psoriatic disease [75].

Conversely, CCL27, a cutaneous T-cell attracting chemokine, is found downregulated in lesional psoriatic skin via IL-17 and IFNγ partially through JNK regulation of cyclooxygenase-2 (COX-2) [76]. In healthy skin fibroblasts, COX-2 induces the production of prostaglandin E2 (PGE2), which then suppresses immunity by increasing the expression of IL-10 and reducing pro-inflammatory cytokines such as IL-23 and TNFα [77]. Fibroblasts derived from psoriatic plaques were found defective in JNK signaling and PGE2 production in response to IL-1β stimulation, both of which were correlated with reduced COX-2 expression. JNK inhibition with SP600125 reduced IL-1β-mediated COX-2 mRNA levels in normal fibroblasts, indicating that JNK is directly involved in the regulation of COX-2 expression. Together, these findings implicate that defective JNK function in fibroblasts contributes to psoriasis linked to deficient PGE2 function [77].

#### 2.2.4. JNK as an Effector of Neuropeptide-Induced Inflammation

Neuropeptide signals have also been shown to play a role in psoriasis by mediating neurogenic skin inflammation [78]. Calcitonin gene-related peptide (CGRP) is one of the most abundant neuropeptides in human skin and is shown to act as a growth factor to induce human keratinocyte proliferation through a rapid increase in phosphorylation of MAPK signaling kinases including ERK1/2, p38, and JNK [79]. CGRP levels and nerve fibers are elevated in epidermal psoriatic lesions [80,81]. Another neuropeptide, substance P (SP), is increased in lesional psoriatic skin. SP acts in part through JNK signaling to promote inflammation synergistically with IL-33-mediated human mast cell activation, which release vascular endothelial growth factor (VEGF) [80,82]. CGRP and SP are frequently co-expressed, and they both counteract beneficial denervation treatment in a psoriasis mouse model [79,83]. Vasoactive intestinal peptide (VIP) is another neuropeptide strongly associated with psoriasis [80]. Unlike CGRP and SP, VIP induces inflammatory cytokines and VEGF through p38 and ERK but not JNK signaling [84].

#### 2.2.5. JNK Links Gap Junction Defects to Psoriasis

Gap junctions consist of transmembrane proteins called connexins (e.g., Cx43, Cx26) that allow for ions, small molecules, and secondary messengers to pass between cells [85]. Such intercellular communications are important for regulation of cellular proliferation, migration, apoptosis, and inflammatory and immune responses. Mutations in connexins (e.g., Cx43 and Cx26) result in decreased protein stability and phosphorylation and thus loss-of-function and such mutations are associated with psoriasis [86]. The proinflammatory cytokine IL-22 was found to downregulate Cx43 gene transcription and promote keratinocyte proliferation and migration through a JNK-dependent manner [85].

#### 2.2.6. JNK Regulation of Barrier Protein Defects

Epidermal barrier proteins, including filaggrin (FLG) and loricrin (LOR) are often downregulated in lesional psoriatic skin, and their downregulation is in part linked to TNFα-JNK signaling [87]. β-galactosidase binding lectin (Gal3) is an anti-microbial peptide predominantly expressed in the epidermis of normal skin. Gal3 was significantly decreased in imiquimod- and IL-23-induced mouse model psoriatic lesions. Gal3−/− mice exhibited epidermal hyperplasia accompanied by an extensive neutrophil accumulation, increased expression of psoriasis-associated proinflammatory molecules such as IL-1β, IL-22, and TNFα, and reduced expression of differentiation markers such as FLG. The abnormal phenotypes observed in Gal3−/− mice were linked to increased JNK activation [88].

**Figure 3:**
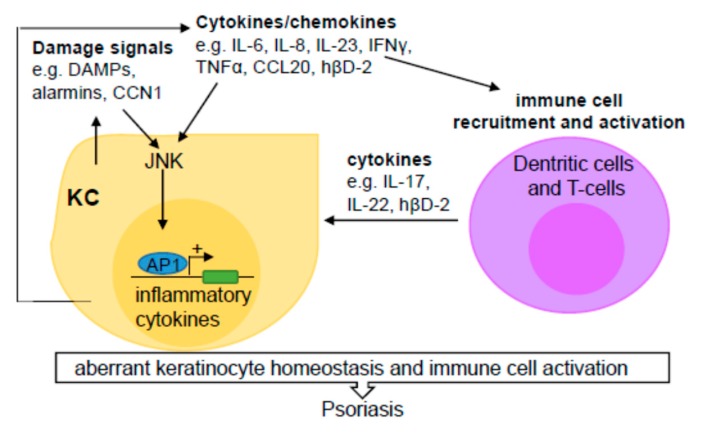
JNK modulates keratinocyte production of inflammatory cytokine/chemokines and recruitment of immune cells in psoriasis. Tissue damage signals (e.g., DAMPs, CCN1) activate the JNK signaling pathway in keratinocytes (KC), resulting in increased expression and release of inflammatory chemokines (e.g., CCL20, and hβD-2) and cytokines (e.g., IL-6, IL-8 IL-23, IFNγ, and TNFα). These molecules not only propagate inflammatory signals in keratinocytes, but also stimulate recruitment and activation of Th1/Th17 immune cells, which produce additional cytokines (e.g., IL-17, IL-22, and hβD-2), leading to propagated dysregulation of keratinocyte proliferation and differentiation and consequently development of psoriasis.

Taken together, JNK mediates keratinocyte cell production and the release of chemokines and cytokines, leading to the recruitment of immune cells. These immune cells stimulate further dysregulation of skin cell proliferation and the continued amplification of the disease state [49,50,69,70,71,72,73] (Figure 3).

### 2.3. Dermal Fibrosis

#### 2.3.1. Pathogenesis of Dermal Fibrosis

The fibrotic response is an integral component of normal wound healing and the repair process; however, the overactivation of the Th2 inflammatory response leads to fibrosis [89]. Scleroderma is an autoimmune disorder characterized by the hardening and tightening of the connective tissues [90,91]. The etiology of scleroderma is complicated. It involves vascular injuries, immune activation, and consequently excessive fibrosis of the skin and internal organs, including lung, gastrointestinal tract, and heart [92,93]. Central to the development and progression of fibrosis is the activation of resident fibroblasts, namely their differentiation into myofibroblasts, resulting in overproduction and impaired degradation of extracellular matrix (ECM) components [93,94,95,96]. Myofibroblast differentiation is initiated by profibrotic cytokines such as transforming growth factor-beta (TGFβ) and platelet-derived growth factor (PDGF) [92,97,98,99,100].

#### 2.3.2. JNK Connections with TGFβ and PDGF in Dermal Fibrosis

The mitogen-activated protein kinases (MAPKs), including JNK, have been linked to the aberrant activation of fibroblasts and subsequent fibrosis [92,101]. JNK is activated by TGFβ and PDGF in Systemic sclerosis (SSc) fibroblasts. Inhibition of JNK by the selective small-molecule inhibitor CC-930 inhibited the release of extracellular matrix proteins by cultured fibroblasts, prevented fibrosis induced by bleomycin, and in tight skin 1 (TSK1) mice, and most importantly induced regression of pre-established fibrosis [92,102].

JNK phosphorylates c-Jun, leading to stabilization of c-Jun and enhanced transactivation activity [103]. pc-Jun is increased in lesional skin biopsies from SSc patients compared with that of healthy control tissues and the increased staining was particularly observed in fibroblasts and the endothelial cells of small blood vessels of SSc samples [104]. JNK inhibition using CC-930 reduced the stimulatory effects of TGFβ and PDGF on c-Jun phosphorylation [92]. c-Jun activity was elevated in human SSc lesional skins, as well as mouse lesional skins induced by bleomycin or adenovirus-mediated expression of a constitutively active TGFβ receptor type I protein [104]. Similarly, pJNK is expressed at an elevated level in monocytes and neutrophils of scleroderma tissues compared to normal tissues [105]. Conversely, ablation of JNK1 but not JNK2 globally or in airway epithelia resulted in a strong protection from bleomycin and adenovirus-mediated expression of the active TGFβ [106,107]. Further, deletion of the Jnk1 allele in fibrotic skin induced a reversal of the fibrotic phenotype [107].

#### 2.3.3. JNK Connections with STAT3 and WNT Signaling Pathways in Dermal Fibrosis

JNK mediates activation of the signal transducer and activator of transcription 3 (STAT3), which is a member of the STAT protein family implicated in tissue fibrosis [96]. STAT3 signaling is hyperactivated in a TGFβ-dependent manner, and this activation is mediated by the combined actions of JAK, SRC, c-ABL, and JNK kinases in SSc fibroblasts [96]. Immunofluorescent staining detected elevated levels of pJNK in fibroblasts of SSc skin compared to that of healthy skin. Inhibition of JNK by either siRNA-mediated gene knockdown or the small molecule inhibitor SP600125 inhibited TGFβ-induced phosphorylation of STAT3, indicating that JNK plays an important role in TGFβ signaling and fibrosis [96].

JNK also mediates fibrosis driven by the Wnt signaling pathway [106,108,109]. Wnt signaling stimulates the release of collagen via JNK/c-Jun independent of the canonical Wnt/β-catenin signaling [108]. Fibroblasts are the major source of canonical and non-canonical Wnt proteins such as Wnt-1, Wnt-10b, and Wnt-5a in SSc [109,110]. Evenness interrupted (EVI) is a multipass transmembrane protein localized in the Golgi and at the cell surface, and it is essential for secretion of canonical and non-canonical Wnt ligands [109,111]. Knockdown of EVI in fibroblasts prevented the release of Wnt ligands, accumulation of β-catenin, and phosphorylation of JNK/c-Jun [109].

#### 2.3.4. JNK Regulation of Extracellular Matrix Proteins in Dermal Fibrosis

Tissue fibrosis is a result of an imbalance of ECM deposition and degradation. TGFβ upregulates type I collagen and TIMP metalloproteinase inhibitor 1(TIMP-1) and downregulates metalloproteinase-1(MMP1) [112]. MMP1 is a collagenase that breaks down interstitial and type I, II, and III collagens and critical for ECM remodeling [113,114]. JNK/AP1 (c-Fos/c-Jun), along with other MAPKs and NF-κB, is crucial for IL-17 and rapamycin-induced MMP1 production in human dermal, cardiac, and lung fibroblasts [94,115,116]. JNK inhibition by SP600125 prevented the upregulation of MMP1 by rapamycin and UVB in SSc dermal fibroblasts [93,117]. While AP1 is required for MMP1 expression in SSc fibroblasts, AP1 inhibition with the small molecule compound T-5224 is found to increase MMP1 mRNA levels in fibroblasts derived from healthy individuals [104]. However, another study showed that JNK inhibition in normal human dermal fibroblasts prevented UVB-induction of MMP3, which promotes activation of other MMPs, including MMP1 and pro-MMPs and degradation of type I collagen [117]. Another cytokine linking JNK to fibrosis is monocyte chemoattractant protein 1 (MCP-1, also known as CCL2), which is produced by SSc fibroblasts and promotes the induction of MMP1 [118]. The secretion of MCP-1 is dependent on JNK-mediated signals and regulated by proteasomal degradation [118].

Besides actions downstream of TGFβ, JNK augments TGFβ gene transcription, induces expression of enzymes responsible for activation of the latent form of TGFβ, and directly phosphorylates SMAD3, leading to enhanced transcription of pro-fibrotic molecules [119]. Consistently, blocking JNK activation suppresses TGFβ1-induced fibrosis and inflammation [120].

**Figure 4:**
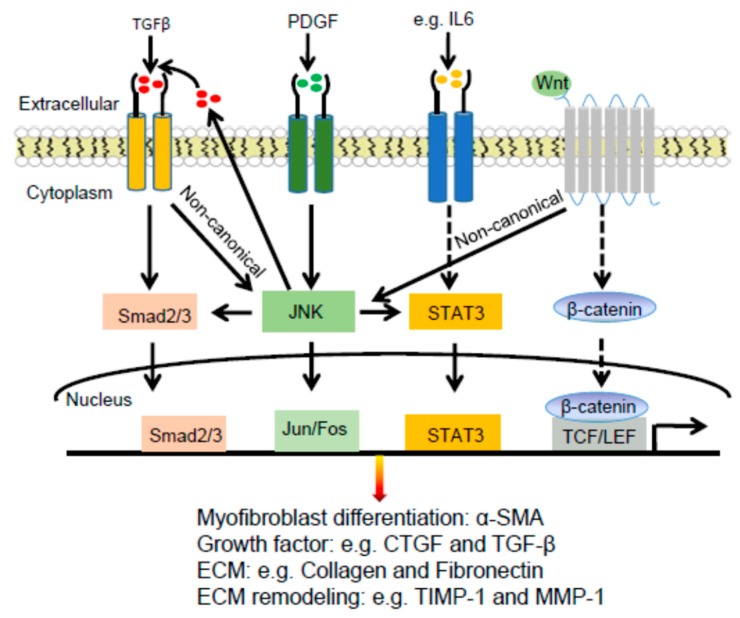
JNK enhances fibrosis via crosstalk with TGFβ, PDGF, STAT3, and WNT signaling pathways. JNK acts downstream of TGFβ, PDGF, and Wnt signaling pathways to regulate expression of profibrotic genes. In addition, JNK enhances TGFβ secretion, and crosstalk with STAT3 to further enhance pro-fibrosis. The dashed lines show the canonical STAT3 and WNT signaling pathways which are not discussed in the review.

In summary, JNK is a common mediator of pro-fibrotic signals, including TGFβ, PDGF, STAT3, and Wnt signaling pathways (Figure 4). While JNK is a potential therapeutic target for the treatment of fibrotic diseases such as scleroderma [92], further studies are needed to characterize JNK subunit and cell type-specific effects on the pathogenesis of fibrosis and immunological reactions.

## 3. JNK Signaling in Skin Cancer

Basal cell carcinoma (BCC) and squamous cell carcinoma (SCC) represent the first and the second most common skin cancers [121,122]. Between 1976–1984 and 2000–2010, the overall incidence of BCC and SCC was increased by 145% and 263%, respectively [123]. Approximately 3 million cases of BCC and SCC were diagnosed in the US in 2019 [124,125]. Melanoma is the fifth most common cancer in men and the sixth most common cancer in women [126]. An estimate of 192,310 new cases of melanoma was diagnosed in the US in 2019, with about 50% of them being invasive [125,127]. Common risk factors for skin cancer include ultraviolet (UV), ionizing radiation, arsenic exposure, viral infection, and wounding [128,129,130,131,132]. JNK, as a dominant responder of these environmental stimuli, plays paradoxical roles in cancer development with both oncogenic and tumor suppressor properties [133,134].

### 3.1. Differential Roles of JNK1 and JNK2 in SCC

JNK activation is frequently observed in SCC [135,136]. Specifically, JNK2 phosphorylation is increased in SCC cell lines and tissues compared to normal keratinocytes and healthy skin samples, respectively [135,137]. Jnk2 deficient mice were resistant to skin cancer development following induction by the DMBA (7,12-dimethylbenz[α]anthracene)/TPA (12-O-tetradecanoylphorbol-13-acetate) two-stage carcinogenesis protocol, indicating that JNK2 functions as a promoter of skin cancer [138]. Consistently, compared to WT mice, Mkk4 deficient mice displayed significantly reduced numbers of skin tumors after 20 weeks of DMBA/TPA treatment, which was attributed to reduced JNK2 activity [139]. In contrast to JNK2, JNK1 showed a tumor suppressor function. Jnk1 deficient mice displayed a higher papilloma incidence than that of wild-type mice [140]. In agreement with these findings, constitutively active MKK7 and MKK7-JNK2 fusion proteins, but not MKK7-JNK1, are able to couple with the oncogenic Ras(V12) to transform normal keratinocytes into SCC-likes lesions [139] and this required intact c-Jun-function [135] and this required intact c-Jun-function [137]. In addition, c-Jun but not JunB can couple with Ras to induce epidermal malignancy [141]. Lastly, squamous cell carcinoma antigen 1 (SCCA1) prevents keratinocytes from apoptotic cell death through inhibition of JNK1 [142]. These data indicate that MKK7, JNK2, and c-Jun, but not JNK1 and JunB, promote epidermal malignancy.

Epidermis-targeted expression of a catalytically deficient CYLD mutant (CYLDm) in K14-CYLDm transgenic mice increased JNK activation and lysine-63 (K63)-ubiquitination and phosphorylation of c-Jun and c-Fos transcription factors [143]. After DMBA/TPA treatment, K14-CYLDm mice developed increased numbers of papilloma, with 66% of them developed into SCC and metastasis by week 32. Topical treatment of the JNK inhibitor SP600125 significantly reduced DMBA/TPA-induced tumor incidence and abolished skin cancer metastasis to lymph nodes in K14-CYLDm mice [143]. KDM4A is a demethylase that specifically demethylates the Lysine 9 and 36 residues of histone H3. In correlation with increased KDM4A expression, c-Jun, and FOSL1 (Fra1), protein levels were increased in metastatic human SCC tissues compared to primary SCC tissues [144]. Further, FRA1 was found to enhance head and neck SCC cell proliferation and migration in a c-Jun-dependent manner [145].

### 3.2. JNK as a Key Mediator of the SHH, YAP, and WNT Signaling Pathways in BCC

The sonic hedgehog (SHH)/Gli signaling pathway plays a dominant role in BCC [146]. JNK inhibition with SP600125 and siRNA knockdown of c-Jun inhibited Gli-induced cell cycle progression, indicating that JNK and c-Jun are important for Hedgehog (HH)/Gli-driven BCC [147,148]. In HaCaT keratinocytes, increased JNK expression was linked to the BCC-like phenotype induced by SHH expression [149]. Interestingly, another study showed that the SHH/Gli signaling pathway acts in synergy with the epidermal growth factor receptor (EGFR) to promote BCC, which requires c-Jun activation by MEK/ERK, but not JNK [150]. In addition, c-Jun and Fos transcription factors interact with phosphorylated ATF2, and are required for ATF2-driven transformation of epidermal cells into BCC [151,152]. Moreover, in a BCC tumor model generated via subcutaneous injection of TetON inducible CRISPR-Yap ASZ mouse cells into immunocompromised (nu/nu) mice, it was found that, after one-week treatment of Doxycycline, the Yap null tumors displayed reduced pJNK1/2 and pJun(S63/S73) levels compared to those of WT BCC tumors [153]. In addition, c-Jun mRNA was significantly decreased in YAP-negative BCC clones and BCC cells treated with SP600125. Lastly, WNT16B, a member of the WNT gene family, was found upregulated in BCC tissues s and its increased expression enhanced proliferation of primary and immortalized human keratinocytes in a JNK-dependent manner [154]. Taken together, these data indicate that the JNK signaling pathway is a critical mediator acting downstream or in collaboration with SHH, YAP, and WNT signaling pathways to promote BCC [153,154].

### 3.3. Melanoma

#### 3.3.1. JNK1 and JNK2 in Melanoma Growth and Progression

The JNK/AP1 axis is commonly activated in benign and malignant melanoma, and promotes melanoma cell proliferation and invasion [148,155,156,157,158]. One study showed that JNK is activated in over 75% benign nevi and it was predicted to have a role in restricting uncontrolled cell proliferation or survival. During tumor progression, activation of JNK is associated with cell proliferation and shorter relapse-free period for patients with superficial spreading melanomas [155].

siRNA-mediated silencing of JNK1 and JNK2 abolished WM164 melanoma cell proliferation, invasion, and metastasis [159]. Likewise, JNK1-specific gene silencing inhibited the growth of melanoma cell lines with high levels of pJNK1 expression [160]. In accordance with these findings, JNK activation is increased in the majority of malignant melanoma cell lines, and tissues examined, which was correlated with decreased expression of CYLD [161]. Restoration of CYLD expression in metastatic human melanoma cell lines inhibited melanoma xenograft growth in the skin and lung following subcutaneous and tail-vein injections, respectively. Coexpression of a constitutively active MKK7 or c-Jun mutant overcame CYLD-inhibition of melanoma growth and metastasis [161]. JNK/c-Jun also mediates melanoma cell proliferation and motility driven by MALT1 [162]. Overexpression of Urothelial Cancer Associated 1 (UCA1) inhibited the CREB/MITF/c-Jun melanogenesis axis, while gene silencing of UCA1 activated it [163]. In a murine melanoma model, it was demonstrated that PRDM5 (PRDI-BF1 and RIZ domain-containing) promoted melanoma proliferation and invasion through the upregulation of JNK [164]. SHARPIN is a tumor-associated gene upregulated in many cancers [165,166,167,168]. SHARPIN increases Ras-related protein 1 (Rap1) via JNK/c-Jun and p38 and promotes A375 and A2058 melanoma migration and invasion [169].

To investigate the role of JNK1 and JNK2, one group expressed wild type (WT) and C116S JNK1/2 mutants in melanoma cell lines, and it was found that JNK inhibition with the small molecule agent JNK-IN-8 enhanced proliferation and invasion in cell culture and subcutaneous xenografts expressing JNK2(C116S) mutant [170]. Furthermore, JNK activation was observed in the melanoma cells overexpressing JNK1WT, JNK2WT, and JNK1C116S but not JNK2C116S [170]. These data indicate that JNK2 is required for melanoma malignancy and resistance to BRAF inhibition [170].

#### 3.3.2. Paradoxical Roles of JNK in Melanoma Cell Survival, Apoptosis, and Therapy

JNK/c-Jun plays a crucial role in melanoma resistance to therapies [171]. Specifically, c-Jun promotes melanoma de-differentiation and production of inflammatory cytokines via interaction with MITF and by recruiting immune-suppressive myeloid cells into the tumor microenvironment [171]. shRNA-mediated gene silencing of c-FLIP, a Fas-associated death domain-like interleukin-1β-converting enzyme (FLICE)-like inhibitory protein, inhibits proliferation of A875 malignant melanoma cells, which is attributed to reduced JNK phosphorylation [172]. Inhibition of IL-1β in IL-lβ-positive melanoma cells induced cell growth arrest, which was accompanied by reduced pJNK expression [173]. Immunohistochemistry of stage III melanomas showed that 75% of the 51 cases had strong expression of JNK, 20% had positive expression of pJNK, and all pJNK-positive tumors were IL-1β-positive. siRNA-mediated gene silencing of IL-1β decreased pJNK in A375 and WM793 melanoma cells without affecting total JNK levels, confirming that JNK is activated by IL-1β in melanoma [174].

JNK/AP1 pathway is important in adaptive responses to the MEK inhibitor vemurafenib [164]. JNK inhibition with the small molecule compound BI-78D3 induced apoptosis and inhibited cell growth of BRAF inhibitor-naive and resistant melanoma cells [175]. Melanoma cells treated with the PAK inhibitor PF-3758309 displayed increased activation of JNK, β-catenin, and the mTOR signaling pathway and shRNA-mediated gene silencing JNK and β-catenin further decreased melanoma cell viability [176]. In addition, JNK pathway inhibition sensitized BRAF mutant melanoma cells to genetically modified vaccinia virus-mediated cell death [163].

Paradoxically, JNK is also found to induce melanoma cell apoptosis. Quercetin, a plant-derived polyphenol compound, induced apoptosis of A375SM and A375P melanoma cells by increasing pJNK expression both in vitro and in vivo [168]. Similarly, treatment of human SKMel-188 melanoma cell line with *Coriolus versicolor fungus*-derived protein-bound polysaccharides induced apoptosis, and increased ROS levels, both of which were inhibited by SP600125 [169].

**Figure 5:**
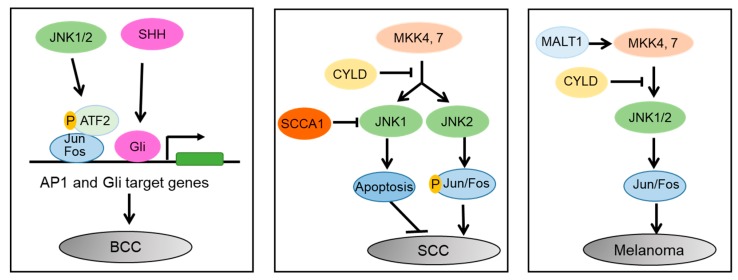
Differential roles of JNK1 and JNK2 in skin cancers. In BCC, JNK1/2 activates Jun/Fos, and enhances their interaction with phosphorylated ATF2, which then enhances SHH/Gli induced tumorigenesis. In SCC, MKK4/7 activates JNK1 and JNK2. JNK1 induces apoptosis, whereas JNK2 promotes carcinogenesis in an AP1-dependent manner. SCCA1 promotes SCC via inhibition of JNK1 and CYLD inhibits SCC via suppression of JNK2/AP1 cascade. In melanoma, the MALT1, MKK4/7, and JNK/AP1 signaling cascade promotes melanoma cell proliferation and migration, whereas CYLD inhibits it.

In summary, JNK proteins play important and distinct roles in different skin cancers (Figure 5). In BCC, JNK1/2 activates Jun/Fos, and enhances their interaction with phosphorylated ATF2, which then enhances SHH/Gli induced tumorigenesis. In SCC, MKK4/7 activates JNK1 and JNK2. JNK1 induces apoptosis, whereas JNK2 promotes carcinogenesis in an AP1-dependent manner. SCCA1 promotes SCC via inhibition of JNK1, while CYLD inhibits SCC via suppression of JNK2/AP1 cascade. In melanoma, the MALT1, MKK4/7, and JNK/AP1 signaling cascade promotes melanoma cell proliferation and migration, whereas CYLD inhibits it.

## 4. JNK as a Therapeutic Target

JNK has been recognized as a potential therapeutic target for many diseases. A number of peptides and small molecule inhibitors have been developed to either directly target JNK [136,165,166,167] or indirectly through inhibition of canonical and non-canonical JNK activators [168].

The ATP-competitive JNK inhibitors include small molecules from various scaffolds such as indazoles, pyridine carboxamides, aminopyrazoles, aminopyridines, benzothien-2-ylamides, benzothiazol-2-yl acetonitriles, quinoline derivatives, and aminopyrimidines [102,169,177,178,179]. Among these, CEP-1347, also named KT7515 or 3,9 bis [(ethylthio)methyl]-K252a, is a derivative from the natural compound K252a. CEP-1347 was found to prevent the death of neurons both *in vitro* and *in vivo* [180]. CEP-1347 induced differentiation and inhibited proliferation of human cancer cells, including glioblastoma (GS-Y01, GS-Y03, and GS-NCC01), pancreatic (PANC-1 CSLC), and ovarian (A2780 CSLC, and TOV21G CSLC) cancer cells [181]. Further, systemic administration of CEP-1347 at 1.5 mg/kg/day for 10 days significantly reduced tumor-initiating cancer stem cells within established tumors and prolonged the survival of mice receiving orthotopic implantation of glioma stem cells [181].

SP600125 is one of the most studied ATP-competitive JNK inhibitors derived from anthrapyrazolone [182]. Its in vivo activity was demonstrated in mouse models of Parkinson’s disease [183] and skin cancer (K14-CYLDm) [143]. SP600125 induced melanoma apoptosis and cell cycle arrest via the induction of p53, Bad, and Bax levels in 1205Lu and WM983B melanoma cells [160]. Another drug, benzothiazolone AS601245, showed neuroprotective effects after focal cerebral ischemia in rats [184] and ischemia-reperfusion injury [185]. JNK-IN-8 is a novel compound that forms a covalent bond between the conserved cysteine in the ATP sites, leading to irreversible inhibition of all three JNK proteins [136]. CC-930 is a potent JNK inhibitor that showed efficacy in inhibiting preclinical models of dermal fibrosis induced by bleomycin and in the tight skin 1 (TSK1) mouse model [92,102]. A phase I clinical study showed that CC-930 was well-tolerated in healthy volunteer patients, and induced a dose-dependent reduction of dermal fibrosis in SSc diseases [186]. The phase II clinical trial of CC-930 in patients with idiopathic pulmonary fibrosis (IPF) showed similar pharmacokinetic parameters to those found in the phase I [187]. Unfortunately, further preclinical trial (NCT01203943) of this compound was terminated due to the increased risk of liver damage [187].

Peptide inhibitors target protein-protein interactions between JNK and substrates such as c-Jun and adaptor proteins such as JIP [188]. D-JNK-1 is a potent and membrane-permeable peptide inhibitor derived from the minimal JNK-binding region of JIP1 [189,190,191]. D-JNK-1 showed a neuroprotective effect on animal models of stroke [180,192]. TI-JIP, another peptide derived from the JNK-binding domain of JIP-1 (amino acids 143–153), showed potent inhibition of JNK activity towards recombinant ATF2, c-Jun, and Elk [190,191].

JNK inhibitors showed promising results in preclinical models, but their clinical benefit has not been appreciated so far. A major challenge with small molecular inhibitors is the non-specific side effects, as they target the highly conserved ATP-binding site, which are present in many different MAPKs. For example, at higher concentrations, SP600125 not only inhibits the three JNK proteins [169], but also affects the closely related ERKs and p38 MAPKs [182,193].

## 5. Conclusions

JNK proteins regulate a multitude of cellular processes, including cell cycle, cell differentiation, cell proliferation, apoptosis, and inflammatory responses. Dysregulation of JNK signaling is inherently linked to psoriasis, skin fibrosis, and non-melanoma and melanoma skin cancers. Nevertheless, our understanding of JNK functions in these diseases is still limited and complicated by the isoform-specific and cell type specific responses. Further studies are needed to address JNK isoform-specific functions in a tissue type-specific manner and to better understand JNK upstream and downstream molecules in various disease settings.
